# Promoting healthy foods in the new digital era on Instagram: an experimental study on the effect of a popular real versus fictitious fit influencer on brand attitude and purchase intentions

**DOI:** 10.1186/s12889-020-09779-y

**Published:** 2020-11-10

**Authors:** Frans Folkvord, Elze Roes, Kirsten Bevelander

**Affiliations:** 1 Open Evidence Research, Barcelona, Spain; 2 Tilburg School of Humanities and Digital Sciences, Tilburg University, Tilburg, the Netherlands; 3 Radboud Institute for Health Sciences, Radboud University and Medical Centre, Nijmegen, the Netherlands; 4 Behavioural Science Institute, Radboud University Nijmegen, Nijmegen, the Netherlands

**Keywords:** Social influencers, Marketing, Healthy foods, Public health, Attitude, Intention

## Abstract

**Background:**

Most studies on social influencer marketing techniques have focused on the promotion of unhealthy foods whereas little is known about the promotion of healthier foods. The present experimental study investigated whether a popular real versus fictitious fit influencer is more successful in promoting healthy food products. In addition, we examined the role of parasocial interaction as an underlying mechanism of healthy food product endorsement.

**Methods:**

We used a randomized between-subject design with 154 participants (mean age: 24.0 years). Viewers’ product attitude and purchase intention were tested after exposure to an Instagram post by a popular real fit influencer (*n* = 77) or fictitious fit influencer (*n* = 77).

**Results:**

Results showed that parasocial interaction mediated the relation between the type of influencer and product attitude as well as purchase intention. Parasocial interaction was higher for participants exposed to the popular real fit influencer compared to the fictitious fit influencer, leading to higher healthy food brand attitude and purchase intention.

**Discussion:**

The findings showed that it is crucial for social influencers to establish a warm personal relationship and connection with the their followers when promoting a healthy product successfully. We suggest that the promotion of healthy foods could be more successful in public health when using popular fit influencers.

## Background

Nowadays, people are continuously exposed to an overload of unhealthy food appeals in their physical as well as online environment [22, 24, 26]. During the last decade, sharing online video content on YouTube and other social media platforms like Facebook and Instagram has become increasingly popular [45]. Following other people on online platforms has become a daily activity among millions of people worldwide [40]. Food advertisers have started collaborations with popular ‘social influencers’ to target their followers by promoting (food) products or services via online social media platforms [8, 10, 14, 17, 22, 24, 28, 30]. Although there is an emerging body of academic research investigating this social influencer marketing technique, most studies have focused on the promotion of unhealthy foods and little is known about the promotion of healthier foods [8, 22, 24].

The exponential growth of social media has given rise to micro-celebrities, such as bloggers and vloggers. These new types of celebrities, so-called ‘social influencers’, have received fame through self-branding. Self-branding is a new strategy whereby social media users use their social media activities to engage in strategic self-presentation to attract attention of a large number of young followers, in particular young people [12, 36]. A new online trend that is designed to inspire viewers towards a healthier lifestyle is called ‘*Fitspiration*’ (i.e., the amalgamation of fitness and inspiration). In general, fitspiration stimulates health and well-being through the promotion of healthy eating, higher levels of exercise and self-care [50]. The overall philosophy of strength and empowerment is one that is strongly emphasized by images that are shared online. Because fitspiration has been positioned as a healthy alternative to the Internet-based trend known as ‘*thinspiration*’ (i.e., amalgation of thin and inspiration), it is highly popular among young people and often used to influence predominantly young women [7, 49]. Instagram is a highly popular platform to share fitspiration images while endorsing healthy products by ‘fit influencers’ [50], which has been used as social medium for the current study.

The social influencer-consumer relation differs from traditional one-way communication forms of expert or celebrity endorsement in magazines and on television, because interaction between the social influencer and consumer is highly important. In addition, influencers are seen as specialists in their community and the match between them and the endorsed product can establish a high level of trust among their viewers and followers [13, 41]. Product endorsements by influencers are perceived as more credible and authentic than regular (commercial) messages of celebrities or advertisers, because viewers are more likely to believe they will receive trustworthy advice or get a genuine opinion about certain brands or products. In this regard, influencer marketing can be seen as a new interactive form of electronic word of mouth advertising (eWOM; [51]). It is suggested that mediated experiences with social influencers tap into more interpersonal processes due to the openness, frequency and reciprocal nature of celebrity endorsement than traditional forms of marketing. However, it is interesting to further investigate if fit influencers are able to effectively promote healthier food products [22].

Therefore, the current experimental study investigated the role of ‘parasocial interaction’ as an underlying mechanism of social influencer endorsement of a healthy food product on Instagram. Previous studies have shown that parasocial interaction enhances the feelings of connectedness and loyalty to the endorsed product and the willingness to consume the brand’s products [11, 24, 28, 30, 37, 38, 53]. Social media platforms stimulate two-way communication between the influencer and viewers, reinforcing parasocial relationships. Parasocial relationships are psychological relationships experienced by media users between them and media personalities (e.g., celebrities or fictional characters) [31, 35]. Viewers identify and feel connected with their favorite media personalities, despite having limited and distant interactions with them. In some cases viewers even perceive warm friendship relations with the media personalities when they get to know the influencer better [9, 34]. In the case of Instagram advertising*,* social influencers often let their viewers be part of their personal life by sharing personal and intimate stories and images. This reinforces the identification process and engagement with the influencer [6]. For example, the residential setting that is often shown in vlogs can further increase identification and bonding with the social influencer.

In addition, viewers often do not recognize advertising in sponsored social media messages compared to traditional forms of advertising, which lowers the probability that they will pro-actively use their skepticism towards the social influencer [10, 30, 32]. And even if viewers recognize advertising in sponsored messages on social media, the extent to which they perceive that they share values, attitudes and perceptions with the social influencer creates a connection or bond between both, leading to more positive attitudes towards the product and increased purchase intentions [16, 19]. The few studies on product endorsement in blogs and YouTube vlogs have shown the positive effect of parasocial interaction relationships on purchase intentions [16, 38]. In this study, we therefore expected that parasocial interaction would increase the likelihood that a product endorsed by a social influencer on Instagram was viewed more positively.

Altogether, social influencers seem to be effective in promoting unhealthy foods through parasocial interaction, but it is yet unclear whether the same effects can be found for the promotion of healthier foods by fit influencers. The current study focused on the promotion of healthy food products by testing whether a popular real fit influencer would be more effective in endorsing a healthy food product compared to an unknown fictitious fit influencer on Instagram. We expected that viewers who were exposed to a popular real fit influencer had a more positive attitude towards the healthy food product and a higher purchase intention than those exposed to a fictitious fit influencer due to higher parasocial interaction.

## Methods

### Design and participants

This study involved a between-participants design (popular real vs. unknown fictitious fit influencer), while testing the mediating role of parasocial interaction on participants’ product attitude and purchase intention of a healthy product. Inclusion criteria were that participants were 18 years or older and followed a real fit influencer on Instagram. Participants were assigned to the conditions randomly. Power calculations were conducted using the program G*Power 3.1.9.4 [20, 21]. To detect a medium to large effect using linear multiple regression, a minimum of 105 participants were needed (f^2^  =  0.20, power .95, *p* < .05).

Participants were recruited via a post by a popular real Dutch fit influencer on Instagram. Of the 246 participants who reacted to her post to participate in research, 92 participants were excluded because they did not complete the study (*n* = 87), were under aged (*n* = 4) or guessed the research aim (*n* = 1). The final sample consisted of 154 participants (*M* age = 24.03, *SD* = 6.05; 94,8% female). The present study was conducted according to the guidelines of the Declaration of Helsinki, and procedures were approved by the Ethics Committee of the Behavioural Science Institute, Radboud University Nijmegen, the Netherlands.

### Procedure

The study took place in May 2019. Participants were followers of a popular real Dutch female fit influencer on Instagram who cooperated in this study. She posted a recruitment message twice via Instagram stories, a feature that lets users post photos and videos which automatically disappear after 24 h. In the recruitment message, the fit influencer asked her followers to participate in a 10-min research survey and provide their opinion and feedback on different Instagram pages with the chance to win one of the three allotted vouchers (€ 10,- each). When the participants ‘swiped up’ (i.e., in Instagram this resembles clicking on a link to direct you to another Internet page), they were forwarded to our official online survey that further informed them about the study.

In a cover story, participants were asked to answer questions about their social media use and evaluate different Instagram pages. All participants were exposed to an Instagram post of an experienced traveler and a news site made up by the researchers. In between these two posts, they were exposed to our manipulation which was either a post of the real or fictitious fit influencer promoting a mock healthy product called ‘*Green Recovery*’. Green Recovery was described as a healthy vegetable cottage cheese – low in carbs and high in proteins – which was excellent to eat after performing exercise and sports. The promotion of such a product fits to the real product range that is often promoted by fit influencers. After the study ended, participants were debriefed about the real aim of the study and were asked to provide active consent to use their data in our study.

### Stimulus material

The participants were exposed to a photo supplemented with text on Instagram in which either the real or fictitious fit influencer endorsed the product Green Recovery. The fit influencers looked alike and were sitting on a bench outside in the sun while eating Green Recovery out of its package. The text next to the Instagram photo contained a message from the fit influencer informing participants that it is important to consume proteins after performing sports and that the influencer ate delicious vegetable cottage cheese of Green Recovery on that day. The fit influencer also added that the cottage cheese is low in calories and has a smooth soft taste. In the other bogus posts before and after the manipulation, viewers were exposed to travel and animal images with text about the magnificent view and animals, respectively.

### Measures

#### Descriptives

Demographic characteristics were assessed consisting of participant’s age, sex, and education level. Additionally, the frequency of participant’s Instagram usage was asked.

#### Product attitude

The attitude towards the promoted product Green Recovery was measured by a 4 item questionnaire adapted from two validated questionnaires [33, 48] ranging from 1 to 10, where 1 was negative and 10 was positive. The questions focused on how much participants liked the product, if they were interested in the product, whether they found the product good for themselves and were attracted to the product (Cronbach’s α = .932).

#### Purchase intention

The intention to buy the product Green Recovery was measured by 4 statements focusing on whether participants wanted to try the product, would search for Green Recovery in the stores and buy the product [3], ranging from 1 (‘completely disagree’) to 6 (‘completely agree’) (Cronbach’s α = .900).

#### Parasocial interaction

Parasocial interaction was measured by a scale from a study that examined vlogger’s influence on consumer perceptions [38, 44]. The scale measures feelings of trust, desire to interact and perceived friendship with the influencer, ranging from 1 (‘completely disagree’) to 6 (‘completely agree’) (Cronbach’s α = .876).

#### Identification

Identification with the influencer was measured with the Inclusion of Other in the Self Scale [2]. The scale exists of seven pairs of circles with the circles representing ‘You’ and the ‘Influencer’. The circles stand apart (1) and come closer each step until they almost overlap (7). Participants were asked to select the pair of circles that best described their relationship with the fit influencer.

#### Congruency

The match between the real or fictitious fit influencer and the endorsed product was measured by the statement ‘The fit influencer and Green Recovery are a good match’ [39]. The answering category ranged from 1 (‘completely disagree’) to 6 (‘completely agree’).

#### Similarity

Attraction to a social influencer has been found to be higher when there is a perceived connection with the influencer [4, 38]. Social attractiveness was measured by an adapted scale for social influencers specifically [38]. There are six statements measuring whether the fit influencer is perceived similar to the viewer, whether the influencer behaves like the viewer, has a lot in common with the viewer, etc. Participants answered to statements on a scale ranging from 1 (‘completely disagree’) to 10 (‘completely agree’)(Cronbach’s α = .876).

### Analysis

First, randomization checks were performed by using one-factor analysis of variance to test for differences among the two experimental intake conditions. Second, Spearman’s rank and Pearson’s correlations were performed for the model variables of age, sex education and time spent on Instagram to determine which variables had to be controlled for in the main analyses. Data analyses were conducted in SPSS for Windows (version 22.0, 2012, SPSS Inc., Chicago, IL, US). Third, we tested whether our manipulation was successful by comparing the antecedents identification, congruency and similarity of parasocial interaction between the real and fictitious condition. This was based on the model proposed by Lee and Watkins [38] in which they showed that parasocial interaction was associated with these antecedents. Finally, for our main analyses, to investigate whether parasocial interaction mediated the relation between a real and fictitious fit influencer on product attitude and purchase intentions, a path model (with parasocial interaction, product attitude and purchase intentions as latent variables) was tested using bootstrapped standard errors (1000) and estimator ML [42] (Mplus Version X; Muthén and Muthén, 1998–2017). Model fit information was assessed by the following fit indices: the χ2 test of model fit, the Comparative Fit Index (CFI) and the Tucker-Lewis Index (TLI; cut-off values close to or above .90). The model provided acceptable fit to the observed data with χ2 test of model fit being significant (*p* < .0001) and according to the values for CFI (.92) and TLI (.90).

## Results

### Descriptives

Most participants were female (94.8%) varying from 18 to 54 years old, of which 61% was between 18 and 23 years old. The education level of the participants was mixed with participants who (had) followed middle education (54.5%), lower education (30.5%) or higher education (22.1%). Of the sample, 20.7% reported to spend less than 30 min per day on Instagram, 37.7% of the sample reported to spend 30–60 min per day and 43.5% reported to spend even more time per day on Instagram (32.5% 1–2 h and 11% more than 1–2 h).

Spearman’s and Pearson’s correlations were performed between these variables and product attitude and purchase intentions. Only product attitude and purchase intentions were correlated significantly (*r*_s_ = 406, *p* < .001). Randomization checks (Table 1) showed there were no differences between conditions. Based on the correlations and randomization check, product attitude and purchase intentions were tested in the same path model without covariates.

**Table 1:** Randomization checks of variables measured by experimental condition^a^

Variables	Fictitious fit influencer (***n*** = 77)	Real fit influencer (***n*** = 77)	***P***-value^**b**^
Age (y)	23.44 (5.18)	24.61 (6.79)	.231
Sex (*n*/*n*)	3/74	5/72	.468
Education level (lower/middle/higher)	15/42/20	21/42/14	.525
Time spend on Instagram (< 1 h/1-2 h/> 2 h p/d)%)	67.5/35.1/10.4	58.5/29.9/11.7	.250

^a^Values are in means ± SDs, minimum–maximum; ^b^Reflects the differences in total means between conditions by one-factor ANOVA or Pearson’s chi square test

### Manipulation check

The manipulation checks showed that there were significant differences between the popular real and unknown fictitious fit influencer conditions on identification (*F*_1,153_ = 40.348, *p* < .001), congruency (*F*_1,153_ = 7.275, *p* = .008) and social attractiveness (*F*_1,153_ = 4.574, *p =* .034). This means that the manipulation was successful because participants felt more connected and similar to the popular real fit influencer and reported a greater perceived match between the influencer and the endorsed product compared to those exposed to the product endorsement by the fictitious fit influencer (see Table 2).

**Table 2:** Manipulation checks for the variables identification, congruency and similarity^a^

Variables	Fictitious fit influencer (***n*** = 77)	Real fit influencer (***n*** = 77)	***P***-value
Identification	1.180 (1.448) (0–5)	2.740 (1.593) (0–7)	<.001
Congruency	4.390 (.934) (1–6)	4.770 (.793) (2–6)	.008
Similarity	5.566 (1.727) (1–9)	6.197 (1.930) (1–10)	.034

^a^Means ± (SD), min-max

### Main analysis

The path model showed that the fit influencer condition significantly predicted parasocial interaction (*B* = .965, *SE_B* = .197, *p* = < .001, 95% CI [.593,1.358]) and that parasocial interaction significantly predicted product attitude (*B* = .472, *SE_B* = .204, *p* = .021, 95% CI [.062,.843]) as well as purchase intentions (*B* = .774, SE = .132, *p* < .001, 95% CI [.520,1.021]). This means that the popular real fit influencer was associated with higher parasocial interaction than a fictitious fit influencer, leading to higher product attitude and purchase intentions. The indirect effects were significant for product attitude (*B* = .455, *SE_B* = .222, *p* = .040, 95% CI [.081,.961]) and purchase intention (*B* = .747, *SE_B* = .195, *p* < .001, 95% CI [.422, 1.197]). Importantly, the direct effect between the fit influencer condition and purchase intentions remained significant, meaning that there was a partial mediation effect (*B* = −.710, *SE_B* = .238, *p* = .003, 95% CI [− 1.162, −.242]).

## Discussion

The current study was the first to test whether promoting healthy foods by a popular real fit influencer on the social media platform Instagram led to a higher product attitude and purchase intention compared to the promotion of healthy foods by an unknown fictitious fit influencer. The study showed that it is crucial for influencers to establish a warm personal relationship and connection (i.e., parasocial interaction) with the their followers to promote a healthy product successfully.

In line with our expectations, we found that viewers who were exposed to the popular real fit influencer had a more positive attitude towards the healthy food product and a higher purchase intention than those who were exposed to the fictitious fit influencer due to higher parasocial interaction. Our findings are in line with the parasocial interaction theory which explains that repeated exposure to a media personality increases people’s feelings of friendship and trust with that personality [35]. Consequently, higher feelings of identification, congruency and similarity, which are related to parasocial interaction relationships, resulted in a more positive product attitude and purchase intention [13, 38].

Our findings also showed that there was not only a relationship between parasocial interaction and the purchase intention of the endorsed product, but also a direct relationship between the popular real fit influencer and purchase intention (i.e., partial mediation effect). Research has shown that people’s consumption behavior is strongly influenced by one’s direct (online) environment [22], thereby particularly emphasizing on the interaction between personal and environmental factors [5, 47]. According to the *Social Learning Theory* [5] people acquire cognitions and behaviors from their social agents through the process of modeling, reinforcement, and social interaction. At the moment, young people spend an enormous amount of their time on social media platforms [1], increasing the importance of enhancing our understanding of the influences of online media behavior.

Therefore, it has been suggested that online celebrities (i.e., social influencers) have a strong impact on consumer socialization, because they are considered as peers and layman and thus more credible than traditional celebrity endorsers [18]. Considering we found an effect on purchase intention and not for product attitude, it could mean that the viewers considered the influencers as highly trustworthy, thereby not activating any form of skepticism when exposed to the images. Viewers did not seem to reflect on whether they even liked the product, but simply followed the recommendations of the fit influencer and wanted to buy the products that were promoted, which is in line with the Processing Commercial Media Content (PCMC) model [10]. This model suggests that when advertising is more integrated in the content of the media message, mainly entertaining content, it is difficult to activate skepticism because the advertising message is not recognized as such.

Due to the rapid developing forms of communications that youth currently use to share their daily experiences, food marketers have realized that collaborations with social influencers to target followers and promote their (food) products or services on these online platforms is essential to keep selling their brands and products [8, 10, 14, 17, 24, 49]. This study has added to the existing knowledge that the same mechanism can be applied for healthier foods, showing that fit influencers have potential to promote healthier foods among young people.

Current findings are in line with the *Promotion of Healthy Food Model* [22], that states that by increasing attention toward healthier foods through food promotion and reinforcing its value, other people increase their consumption. Social influencers are well suited to create positive associations, increase liking, and act as role models through parasocial interaction. Subsequently, a reciprocal relation with eating behavior occurs, which in time could lead to a normalization of intake of healthy foods. Therefore, health interventions could benefit from utilizing social (fit) influencers, considering their large influence on an important target group [52]. For example, social network interventions utilizing peer influence (i.e., social influencers within school classes) are believed to affect normative behaviors [46]. Research has shown that people do not like to deviate from group norms, which triggers them to conform to the normative behavior of social influencers [15]. When social influencers show and promote a behavior on Instagram, such as the popular real fit influencer in this experimental study, and followers see a large group of other followers liking their product endorsements, they can improve the perceived value of healthier foods and set and change the norm of the type and amount of foods or drinks people should consume.

One of the strengths of the current study is that we examined the influence of a popular real fit influencer. Second, we examined whether this influencer was effective in promoting healthier foods, where food marketing research has predominantly focused on the influence of food advertising of unhealthy foods on eating behavior among children and adolescents [8, 22, 24, 27]. Research examining the effects of food marketing for healthy foods is scarce [22, 23, 25, 27–30]. Considering that a great number of people do not consume enough healthy foods and overconsume unhealthy foods [43], it is of great importance to examine the potential of food promotion techniques of healthier foods. Third, the expenditure of food companies to advertise their brands and products through social influencers is increasing, while the understanding of its effects on eating behavior is lacking. Taking into account that new generation mostly communicate through online platform.

A limitation of the current study is the sample size which could reduce the external validity of the findings. Second, we did not take into account actual consumption. Therefore, it would be interesting for future research to include the intake of the promoted products. For example, to see whether promotion on social media platforms for healthy foods increase the intake of healthier foods such as fruit and vegetables. Another limitation is that primarily young female adults participated, so it is difficult to conclude if the same effects would have been found among other target groups, such as men or elderly. Important to note is that we used a female fit influencer, while a male fit influencer would be more likely to reinforce parasocial interactions and be more effective in promoting healthier foods.

## Conclusions

The current study showed that it is crucial for social (fit) influencers to establish a strong connection with their followers in order to effectively convey their message, for example, promoting healthy food products. New and innovative methods to promote fruit and vegetable consumption are necessary to improve people’s dietary intake [22]. Health interventions could benefit from utilizing social influencers, considering their large influence on an important target group. Future research should investigated whether and how social and fit influencers can be involved in health campaigns.

## Data Availability

The datasets used and/or analysed during the current study available from the corresponding author on reasonable request.

## Abbreviations

CFI: Comparative Fit Index
eWOM: Electronic word of mouth
PCMC: Processing commercial media content
TLI: Tucker-Lewis Index
